# Monte Carlo Simulation and Reconstruction: Assessment of Myocardial Perfusion Imaging of Tracer Dynamics With Cardiac Motion Due to Deformation and Respiration Using Gamma Camera With Continuous Acquisition

**DOI:** 10.3389/fcvm.2022.871967

**Published:** 2022-07-13

**Authors:** Yoonsuk Huh, Uttam M. Shrestha, Grant T. Gullberg, Youngho Seo

**Affiliations:** ^1^Department of Radiology and Biomedical Imaging, University of California, San Francisco, San Francisco, CA, United States; ^2^Molecular Biophysics and Integrated Bioimaging Division, Lawrence Berkeley National Laboratory, Berkeley, CA, United States; ^3^Department of Nuclear Engineering, University of California, Berkeley, Berkeley, CA, United States

**Keywords:** myocardial perfusion imaging, dynamic SPECT, cardiac motion, motion correction, continuous acquisition

## Abstract

**Purpose:**

Myocardial perfusion imaging (MPI) with single photon emission computed tomography (SPECT) is routinely used for stress testing in nuclear medicine. Recently, our group extended its potential going from 3D visual qualitative image analysis to 4D spatiotemporal reconstruction of dynamically acquired data to capture the time variation of the radiotracer concentration and the estimated myocardial blood flow (MBF) and coronary flow reserve (CFR). However, the quality of reconstructed image is compromised due to cardiac deformation and respiration. The work presented here develops an algorithm that reconstructs the dynamic sequence of separate respiratory and cardiac phases and evaluates the algorithm with data simulated with a Monte Carlo simulation for the continuous image acquisition and processing with a slowly rotating SPECT camera.

**Methods:**

A clinically realistic Monte Carlo (MC) simulation is developed using the 4D Extended Cardiac Torso (XCAT) digital phantom with respiratory and cardiac motion to model continuous data acquisition of dynamic cardiac SPECT with slowly rotating gamma cameras by incorporating deformation and displacement of the myocardium due to cardiac and respiratory motion. We extended our previously developed 4D maximum-likelihood expectation-maximization (MLEM) reconstruction algorithm for a data set binned from a continuous list mode (LM) simulation with cardiac and respiratory information. Our spatiotemporal image reconstruction uses splines to explicitly model the temporal change of the tracer for each cardiac and respiratory gate that delineates the myocardial spatial position as the tracer washes in and out. Unlike in a fully list-mode data acquisition and reconstruction the accumulated photons are binned over a specific but very short time interval corresponding to each cardiac and respiratory gate. Reconstruction results are presented showing the dynamics of the tracer in the myocardium as it continuously deforms. These results are then compared with the conventional 4D spatiotemporal reconstruction method that models only the temporal changes of the tracer activity. Mean Stabilized Activity (MSA), signal to noise ratio (SNR) and Bias for the myocardium activities for three different target-to-background ratios (TBRs) are evaluated. Dynamic quantitative indices such as wash-in (K_1_) and wash-out (k_2_) rates at each gate were also estimated.

**Results:**

The MSA and SNR are higher with higher TBRs while biases were improved with higher TBRs to less than 10%. The correlation between exhalation-inhalation sequence with the ground truth during respiratory cycle was excellent. Our reconstruction method showed better resolved myocardial walls during diastole to systole as compared to the ungated 4D image. Estimated values of K_1_ and k_2_ were also consistent with the ground truth.

**Conclusion:**

The continuous image acquisition for dynamic scan using conventional two-head gamma cameras can provide valuable information for MPI. Our study demonstrated the viability of using a continuous image acquisition method on a widely used clinical two-head SPECT system. Our reconstruction method showed better resolved myocardial walls during diastole to systole as compared to the ungated 4D image. Precise implementation of reconstruction algorithms, better segmentation techniques by generating images of different tissue types and background activity would improve the feasibility of the method in real clinical environment.

## Introduction

Non-invasive myocardial perfusion imaging (MPI) using single photon emission computed tomography (SPECT) and positron emission tomography (PET) have been used for diagnosis and prognosis of ischemic myocardial infarction and several coronary artery diseases (1). In conventional SPECT and PET MPI, usually a combined rest and stress study is performed after the radiotracer is fully stabilized in the myocardium. Comparisons of the rest and stress scans thus provide information about ischemic heart disease and myocardial reversibility while the electrocardiogram (ECG)-gated images provide information about the wall motion for the diagnosis of myocardial akinesis and viability. Conventional SPECT MPI has been used as a workhorse to identify patients with coronary artery diseases (CAD) and referrals to a catherization laboratory for possible coronary artery revascularization (2, 3). However, it does not provide information on radiotracer kinetics and hemodynamically significant quantitative information such as uptake rate (K_1_), myocardial blood flow (MBF) and coronary flow reserve (CFR). The quantification of MBF and CFR can detect significant stenotic lesion and microvascular dysfunction. The current state-of-the-art solid-state SPECT MPI technology (4) can provide improved photon sensitivity that has facilitated fast MPI and the feasibility to quantify MBF. Dynamic imaging has been used with PET to quantify MBF (5, 6), and recently some studies have shown that dynamic SPECT imaging can also quantify MBF and CFR (7–12).

This work is inspired by recent activities involving continuous image acquisition and processing with a SPECT camera for myocardial perfusion imaging (13). Basically, the importance of “continuous image acquisition” is to eliminate the dead time between the angles at consecutive detector positions by not acquiring the data which results in loss of significant number of photons. In fact a tomographic step-and-shoot continuous mode acquisition, so-called SwiftScan, has been investigated to determine whether this can be used in clinical practice as a part of time reduction strategy, while preserving image quality and accuracy (14–16). The continuous image acquisition for dynamic scan using conventional two-head gamma cameras provides an additional attribute to MPI, which to our knowledge, has not been initiated yet. The main bottleneck of the dynamic reconstruction with the continuous image acquisition is the inconsistency of the data. For a slowly rotating gamma camera it is difficult to obtain consistent tomographic data to solve dynamic inverse problems frame-by-frame. However, by estimating coefficients of spatiotemporal basis functions directly from projection measurements one can fairly accurately reconstruct the dynamic images and thus estimate the wash-in and wash-out rate parameters of a myocardial perfusion SPECT tracer (7).

The conventional MPI usually results in spatial blurring and motion artifacts due to cardiac and respiratory motion and leads to inconsistency in the tomographic reconstruction that undermines the diagnostic accuracy. Respiratory motion artifacts can be minimized with proper breath holding and shortened scan time or can be partially reduced by ECG gating (17). This method, however, cannot be utilized for dynamic imaging. The cardiac and respiratory motion can be modeled by binning the list-mode (LM) data into projections of different cardiac-respiratory gates. This allows to reconstruct each gate separately (18). This is a powerful reconstruction approach in PET where all projection views can be acquired simultaneously in a LM format and can be used to reconstruct each cardiac or respiratory gate (19). However, this method has not been implemented for SPECT due to limited number of projections and very low camera sensitivity. The incorporation of motion in dynamic imaging with commonly available SPECT system has immense clinical significance. Several methods, mostly applicable for PET system, have been proposed to reduce the motion artifacts (18, 20–26), but cardiac motion is difficult to handle especially when tracer kinetics (wash-in and wash-out) is involved.

In dynamic cardiac SPECT imaging using a slowly rotating gamma camera, photons can be detected and recorded continuously as camera rotates around the subject. Acquiring data in LM format with angle and bin location, in addition to the tags for cardiac and respiratory phases of each photon, allows for more accurate estimation of quantitative measurements and flexibility in modeling the physiological processes of the time varying activity changes with cardiac and respiratory motion. The LM data also allows for retrospective gating and reconstruction to a specific portion of the scan, and thus help to eliminate unwanted artifacts.

A LM maximum-likelihood expectation-maximization (LM-MLEM) iterative reconstruction algorithm can, in principle, provide more accurate image reconstruction than reconstructing the data frame-by-frame. However, reconstruction of LM dynamic data with several tags requires reconstruction of each event and is computationally very challenging. This is particularly difficult to implement in the dynamic reconstruction with the cardiac and respiratory motion for the kinetic parameter estimation when the blood pool is involved.

Recent advent of new dedicated cardiac SPECT cameras has provided a promising avenue for the quantification of MPI and opportunities for the measurement of kinetic parameters, MBF, and CFR non-invasively using either a directly reconstructing parametric images from the projection data (27) or an indirect method of reconstructing the dynamic data frame by frame and then fitting parameters with appropriate compartment model. Both the direct and indirect methods for MPI have been extensively utilized in PET imaging. However, only recently attention has been shifted to SPECT due to improved noise level. Particularly, direct LM parametric reconstruction from the projection data could significantly reduce the noise level. The proposed method emphasizes the importance of “continuous image acquisition” in dynamic cardiac SPECT which eliminates the dead time between the angles of consecutive detector head positions by not acquiring the data which results in loss of a significant number of photons as is done in conventional 3D clinical SPECT.

The stationary dedicated cardiac SPECT camera can acquire data in multiple views without rotating the camera head for dynamic imaging. The main purpose of the proposed method is to extend the static imaging capabilities of the conventional and commonly available dual head SPECT geometry (GE Infinia Hawkeye) to dynamic imaging capabilities with cardiac and respiratory motion correction by acquiring the data in a continuous fashion for quantitative MPI and the method, in principle, can also be applicable to stationary dedicated cardiac SPECT cameras.

In our earlier work, we have demonstrated that it is possible to model all 6 dimensions of space, time, cardiac deformation, and cardiac-respiratory motion from a synthetic data acquired by forward projection using system matrix for a slowly rotating SPECT camera with only a few views per respiratory and cardiac gates (28). Because of the small number of detector heads and the rotation speed of the SPECT camera, it is necessary to obtain the sufficient tomographic views in each cardiac and respiratory state. This can be achieved by increasing the number of views per rotation.

Here, we implemented the 4D maximum-likelihood expectation-maximization (MLEM) reconstruction algorithm (7, 28) for the realistic data generated by MC simulation with the 4D XCAT phantom (29). We extended our earlier work with the data that simulates the clinical continuous acquisition scenario rather than a synthetic data generated by the forward projection of the cardiac torso phantom with the system matrix for the SPECT camera geometry. Unlike in our previously reported 6D spatiotemporal image reconstruction where we have incorporated fully 6D image reconstruction with Gaussian basis functions to model cardiac and respiratory motion (28), here we used the 4D reconstruction for each cardiac and respiratory-gated binned data. Our spatiotemporal image reconstruction uses splines to explicitly model the temporal change of the tracer activity. This method partly follows the development by Verhaeghe et al. for the PET application with spatiotemporal basis functions (30), and for SPECT application (31). Reconstruction results are presented showing the dynamics of the tracer in the myocardium as it moves due to cardiac beating and respiratory motion. These results are then compared with the conventional 4D-spatiotemporal reconstruction method that models only the temporal changes of the tracer activity.

## Materials and Methods

### The SPECT System

In this study, a dual head SPECT camera (GE Infinia Hawkeye) was used to model the activity projection. Monte Carlo (MC) simulations were performed using the GATE (Geant4 Application for Tomographic Emission, version 7.2) (32) for all data acquisitions. Two gamma cameras having 563.2 mm × 563.2 mm × 9.5 mm NaI (Tl) crystals acquired projection data with a radius of rotation of 320 mm. A low-energy high-resolution (LEHR) collimator was coupled with the cameras. Lead shielding on all four sides of each detector was included to avoid scatter events at the side and back compartments representing photomultipliers. Associated electronics were also added to account for potential interactions between emitted photons and photomultipliers. An overall intrinsic spatial blurring of 2.5 mm FWHM was considered at the level of the electronics. A 10% energy resolution at the energy reference of 140 keV for Tc-99m was incorporated (33). Photoelectric effect, Compton scattering, Rayleigh scattering, bremsstrahlung and ionization were also included in the GATE simulation. In order to apply dual-energy window (DEW) scatter correction, scatter and main energy windows were set up in the range of 100 ∼ 125 keV and 126 ∼ 154 keV, respectively, with the scatter multiplication factor of 0.5 (34, 35). Table 1 shows the details of the MC simulation parameters for the SPECT system.

**TABLE 1:** MC simulation parameters for SPECT system modeling.

Parameter	SPECT System
Detector head	2
Detector type	NaI (Tl)-PMT
Detector size (mm^3^)	563.2 × 563.2 × 9.5
Pixel (binning) size (mm^2^)	4.4
Hole shape	Hexagonal
Material	Lead
Hole length (mm)	35
Septal thickness (mm)	0.2
Hole diameter (mm)	1.5
Radius of Rotation (mm)	320

### The Phantom

**FIGURE 1:**
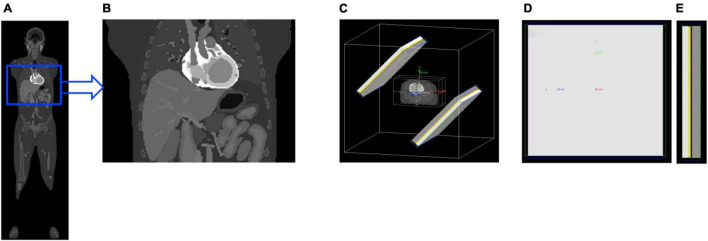
The 4D XCAT whole-body phantom **(A)** was cropped and simplified to model a cardiac torso **(B)**. The torso was then imported to a SPECT system geometry **(C)** with collimator (gray), detector crystal (yellow), back-compartment (white) and lead-shields (blue and green). Also shown are the rear **(D)** and side **(E)** view of the detector.

4D XCAT phantom was used to model the torso part of the human body that included cardiac beating and respiratory motion (36). The original phantom model was established from CT data using Non-uniform Rational B-Splines (NURBS). This simplified torso model has up to 20 materials consisting of myocardium and pericardium, blood pool, ventricles, arteries, veins, muscle, gallbladder, lungs, spleen, stomach, rib bone, cortical bone, spine bone, and liver. These materials were categorized into 4 groups: myocardium, blood, liver, and the rest as background. The matrix size of the original phantom was also modified to reduce the simulation time with particular focus on the cardiac and diaphragm motion. The simplified torso phantom was a matrix of 180 × 120 × 110 elements each corresponding to voxels of 2.2 mm × 2.2 mm × 2.2 mm (Figure 1). Attenuation maps for the same phantom were also generated assuming a Tc-99m isotope at an energy of 140 keV.

**FIGURE 2:**
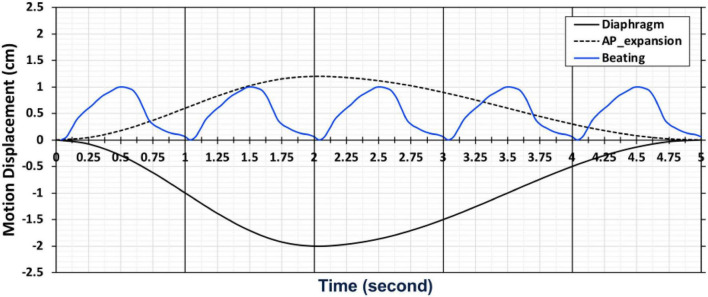
A chart depicting the motion of cardiac and respiratory series for 40 phantoms with 5 cardiac and 1 respiratory cycle from end-inhalation to exhalation.

The beating of the heart and respiratory motions were incorporated in the 4D XCAT phantom. The periods of the cardiac and respiratory cycles were set to be 1 s and 5 s, respectively. The duration between the end-diastolic to the end-systolic phase was fixed at 0.325 s. The extent of anterior-posterior (AP) expansion was 1.2 cm and the displacement due to diaphragm (up and down) motion was initially set to 2.0 cm. The respiratory and cardiac motion were discretized from end-inhalation to exhalation in a series of 40 phantoms each with a duration of 0.125 s corresponding to 8 cardiac gates per heartbeat and 5 heartbeats in each respiratory cycle (Figure 2).

### Time Activity Curves and Data Acquisition

**FIGURE 3:**
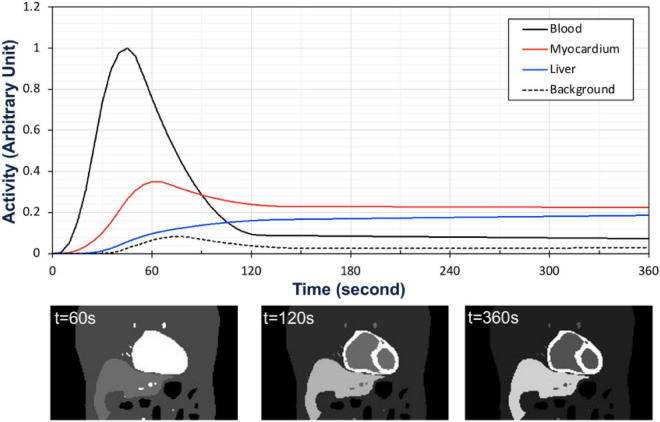
Time activity curves used for the activity map (top) and the corresponding coronal views of the activity distribution in the phantom at time *t* = 60 s, *t* = 120 s, and *t* = 360 s (bottom).

Once the phantom was specified, the activities of different organ tissues such as myocardium, blood, liver and background were simulated using typical time-activity-curves (TACs) of patients in our previously completed dynamic cardiac SPECT studies with Tc-99m tetrofosmin (12). The activity in each tissue in the series of 40 phantoms was changed as a function of time according to the activities in the TACs (Figure 3). Once the activity in the phantom was assigned with a given TAC, the camera was rotated continuously around the phantom acquiring the data at each 0.125 s interval. The rotation speed of the detector heads was adjusted in such a way that the gamma camera acquired the projection data per 0.5^0^ angle increment as the activity in the phantom changes continuously. Therefore, the time per rotation (period) of each camera head was 90 s, which is a typical rotation speed of the GE Infinia Hawkeye SPECT camera. In our simulation, there was a total of 4 rotations per detector head corresponding to the total acquisition time of 6 min.

**FIGURE 4:**
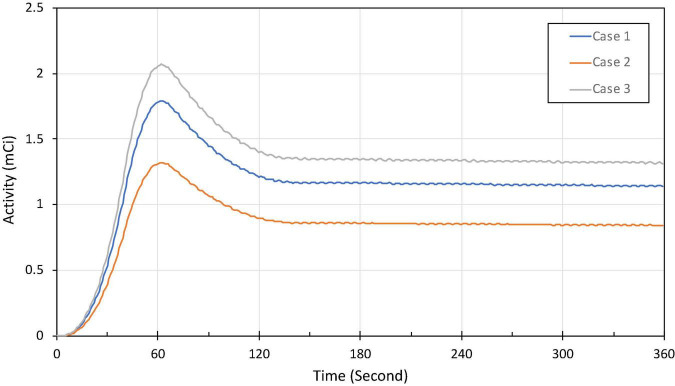
Approximate activity distribution in the myocardium for three different TBRs: 8 (Case 1), 5 (Case 2), and 10 (Case 3).

**FIGURE 5:**
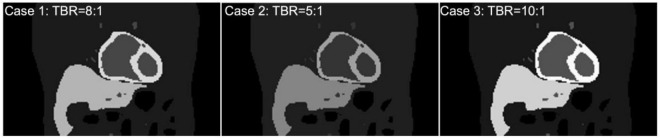
(Top) Coronal view of the activity maps for different TBRs at time *t* = 360 s after the tracer injection (Case 1, 2, 3). All images were normalized by the maximum pixel value of the image with TBR = 10 (Bottom).

To test and validate the reconstruction algorithm, we simulated 5 different cases by modeling the TACs with different target (myocardium) to background activity ratios (TBRs) (Table 2). In all cases, we kept the total activity fixed with approximately 25 mCi at the maximum activity point in the injected dose and varied the ratios of activity of the TBR at 5, 8 and 10 at the end of the acquisition time (Case 1–3). In our simulation we considered Case 1 (TBR = 8) as the reference case for this study. Case 2 with TBR = 5 and Case 3 with TBR = 10 were considered as upper and lower bound of TBR around this reference. We have not explicitly incorporated additional noise in the simulation. However, the lower TBR corresponded to higher background activities which involved higher noise levels. Figure 4 shows the myocardial activities as a function of time for these 3 cases and Figure 5 shows the coronal views of the phantom at the end of the acquisition time (*t* = 360 s) corresponding to these cases.

**TABLE 2:** Simulation cases in this study.

Case	Max. Activity (mCi)	*Myocardium: Background	*Myocardium: Blood	*Myocardium: Liver	LV Thickness	Diaphragm displacement (cm)
1	25.01	8.05:1	3.08:1	1.20:1	Uniform	2.0
2	25.11	5.11:1	3.08:1	1.20:1	Uniform	2.0
3	25.01	10.2:1	3.08:1	1.20:1	Uniform	2.0
4	24.98	8.05:1	3.08:1	1.20:1	Non-uniform	2.0
5	24.99	8.05:1	3.08:1	1.20:1	Uniform	1.5

*Activity ratios at 6 min after injection.

**FIGURE 6:**
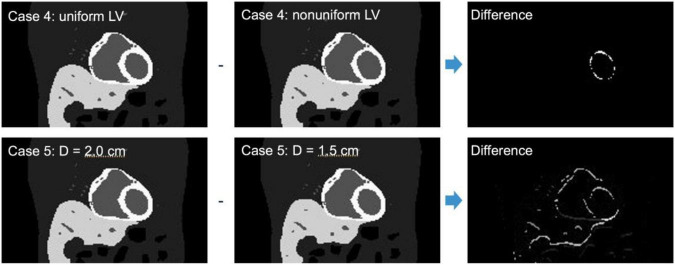
Similar activity map as in Figure 5 for non-uniform LV thickness (Case 4) and uniform LV thickness but with diaphragm displacement of *D* = 1.5 cm (Case 5) and their corresponding differences.

We have also simulated the effect of LV wall non-uniformity (Case 4) as well as the variation in the diaphragm motion (Case 5) for a fixed TBR = 8 (Figure 6). These cases are summarized in Table 2. All simulations were performed in parallel on the high-performance computing clusters in our lab with Xeon (Intel) series CPUs. The output format from the MC simulation can be configured in the fully LM format with information of each photon with the temporal, cardiac-respiratory tag or binned data at a given angle position and the tag of cardiac-respiratory phase. We used binned data with a temporal resolution of 0.125 s in the output of the projection with a detector pixel bin size of 4.4 mm for the reconstruction.

### Spatiotemporal Reconstruction

We modeled the activity distribution of the radionuclide as a function *A*(*x*,*t*,τ(*t*),ζ(*t*)), where *x* is the spatial coordinate, *t* is the temporal coordinate, τ(*t*)is the cardiac phase coordinate due to beating of the heart, and ζ(*t*) is the cardiac displacement coordinate due to the respiratory motion. The activity distribution can be written as a tensor product of the basis functions:


(1) $$A\,\left(x,t,\tau \,\left(t\right),\zeta \,\left(t\right)\right)=\sum_{m,n,q,r}a_{m\,n\,q\,r}\,S^{m}\,\left(x\right)\,V^{n}\,\left(t\right)\,W^{q}\,\left(t\right)\,R^{r}\,\left(t\right),$$

where *Sm*(*x*), *m* = 1, 2,…*M*, are the spatial and *Vn*(*t*), *n* = 1, 2,…*N*, are the temporal basis functions, while *Wq*(*t*), *q* = 1, 2,…*Q*, and *Rr*(*t*), *r* = 1, 2,…*R*, are basis functions corresponding to the cardiac and respiratory phases, respectively. The reconstruction problem thus involves the estimation of the expansion coefficients *a*_*mnqr*_.

For the geometry of our acquisition scheme using continuous rotation shown in Figure 1, the projection of the activity at any particular instant depends on the angular position of the detector. The detector is also pixelated so that for an arbitrary *i*^th^ pixel, the projection of the activity at time point *t* is given by


(2) $$p_{i}\,\left(t,\tau \,\left(t\right),\zeta \,\left(t\right)\right)=\int_{\chi }F\,\left[x,d_{i}\,\left(t\right)\right]\,A\,\left(x,t,\tau \,\left(t\right),\zeta \,\left(t\right)\right)\,dx,$$

where the spatiotemporal distribution of the activity is integrated along the line of projection in the image space χ. The function *F*[*x*,*d*_*i*_(*t*)] maps the activity from a position *x* in the image space into the projection at the detector position *d*_*i*_(*t*), cardiac phase coordinate τ(*t*) due to beating of the heart, and respiratory phase coordinate ζ(*t*).

**FIGURE 7:**
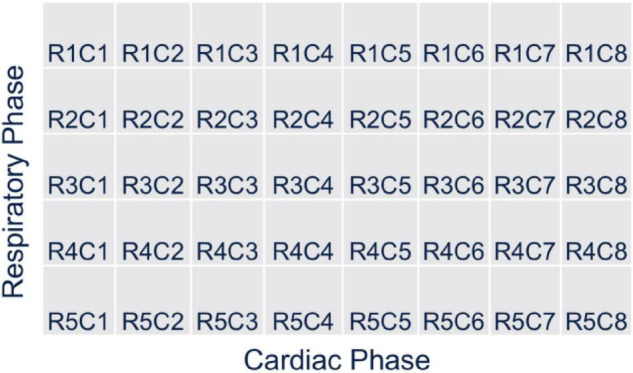
Gating scheme for the reconstruction: There are 8 cardiac and 5 respiratory gates corresponding to 40 gates for each 4D XCAT phantom configuration.

Details of the reconstruction algorithm and its derivation are given in the Ref. (28). The time-varying activity distribution of the radiotracer concentration for each respiratory and cardiac cycle was simulated as described in the previous section. There were 720 projections per rotation per detector head in each simulation and 8 cardiac and 5 respiratory gates corresponding to 40 gates for each 4D XCAT phantom configuration (Figure 7). The rotation of the detector head was adjusted in such a way that the projection time frames were discretized at an interval of 0.125 s per a 0.5^0^ angle increment in a continuous fashion so that the accumulated photons at a given angle position and time were binned corresponding to each cardiac-respiratory phase of the heart as shown in Figure 7. In order to avoid any confusion and preserve the symmetry of the matrix, we have used both the detector bin size in the projection and reconstruction voxel size equal to 4.4 mm.

In our previous study we used an average attenuation map of all 40 phantoms (28). Incorporating the time dependent attenuation map into the system matrix for an “on-the-fly” fully 6D reconstruction would be computationally very costly due to the massive data size. Here, we have generated 40 system matrices corresponding to each gate with a given attenuation map since there were 40 attenuation maps corresponding to each phantom. The attenuation map is the cyclic function of cardiac and respiratory phase. Therefore, there are 18 projections per gate per 360° rotation. The system matrix models the attenuation as well as the geometric point response using the line integral approach developed for the 3D geometry (37, 38), and can be adapted for the 4D model (39). Even though the activity distribution differs greatly with cardiac deformation and respiration, a different system matrix for each cardiac-respiratory phase eliminates the motion artifact in the reconstruction.

**FIGURE 8:**
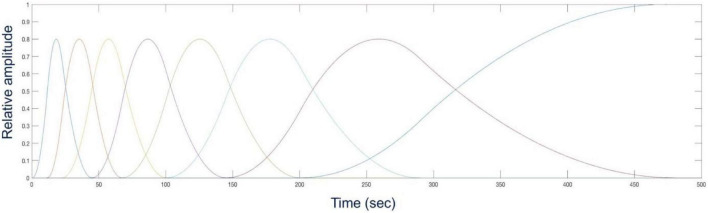
A representative set of temporal cubic B-spline basis functions that were optimized for a specific input time activity curve (TAC) in Figure 3.

The activity distribution was reconstructed for five sets of data with different TBRs to see how the noise in the reconstructed images affects the signal-to-noise-ratio (SNR). There is a trade-off between the number of gates and the number of projections per gate that affects the quality of the reconstructed image (28). However, in the current study we kept the number of cardiac-respiratory gates fixed. In addition to the number of gates all detector parameters were also kept unchanged. The activity distribution in each voxel in the volume changes with time due to not only the temporal changes of the radiotracer distribution but as well as due to the cardiac-respiratory motion. For the gated cardiac-respiratory reconstruction, we modeled the temporal dynamics of the tracer by optimized cubic B-spline basis functions to reconstruct the dynamic time activity of each gate separately. Figure 8 shows a typical set of B-spline basis functions used in the reconstruction.

The scattered correction was implemented in the projections with double energy window. In our reconstruction, we have not incorporated regularization explicitly. However, the use of basis functions in the reconstruction provided some regularization in time (40).

The continuously acquired data was binned for cardiac and respiratory gate and the reconstruction was performed for the binned data set, not for each photon event. It should be pointed out that our reconstruction method, unlike 3D reconstruction, does not directly estimate the activity distribution in the image volume, but solves the coefficients of the basis functions, which are then transformed into a continuous function of activity (Equation 1). The gated reconstructed image provides the information on tracer dynamics for each cardiac respiratory gate thus effectively eliminates the motion artifact. The reconstructed images were then filtered with 4D Gaussian filter (FWHM = 4.5 mm and kernel size = 9).

### Performance Evaluation

To evaluate the performance of our algorithm we compared the SNR and Bias for three different TBRs at 5, 8 and 10. For this purpose, we considered data for only the last minute after the tracer injection with 720 projections per rotation, corresponding to total counts of 2.1×10^6^, 1.8×10^6^ and 2.4×10^6^ for three different TBRs of 8, 5 and 10, respectively. Each view in the detector plane was pixelized with 128 × 128 bins, and the mean photon counts per pixel was 1.2 ensuring the count rate was within the tolerance of the SPECT camera (41). For a given TBR, Mean Stabilized Activity (MSA), SNR and Bias were calculated over the region of interest (ROI). Left Ventricular myocardium was the ROI considered for all performance evaluations. We have also tested the level of significance of the difference between MSA using two-tailed t- test for different TBRs.

### Utility of Dynamic Gated Myocardial Perfusion Imaging

The method described in this study is capable of estimating dynamic quantitative indices such as wash-in (K_1_) and wash-out (k_2_) rates for each gate. These parameters can be estimated by fitting the TAC of the myocardial tissue activity using a compartment model. The myocardial TACs were fitted using a 1-tissue compartment model (1TCM) with LV blood pool as an input function (12) for Cases 1, 2 and 3. The spillover from LV into the myocardium was also corrected by incorporating the blood fraction (V_L_) in the model.

## Results

**FIGURE 9:**
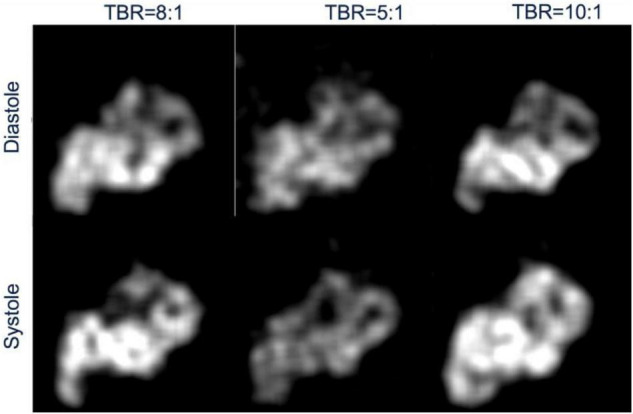
Reconstructed image of myocardial activity distribution during diastole and systole phases of the heart for three different TBRs at full exhalation.

Figure 9 shows an example of the reconstructed image at systolic and diastolic phases for three different TBRs at 6 min after tracer injection. The mean myocardial activity is lower for an initial lower myocardial dose in the phantom, for instance the mean activities at diastole and systole were 0.077, 0.063, 0.10 and 0.089, 0.071, 0.11 for the ratios 8:1, 5:1 and 10:1, respectively, consistent with the ground truth activities. Since the cardiac motion is coined with respiratory phases the systolic and diastolic phases do not represent the same respiratory phase. That means the results presented in Figure 9 do not represent the same image slices.

The sharpness of the image is clearly manifested with no motion blurring. The streak artifact as seen previously in the transverse view in our previous work (28) due to the inconsistency in the projections, with only 12 views per gate, have been eliminated despite the reconstruction was still underdetermined with only 18 views per gate per rotation.

**FIGURE 10:**
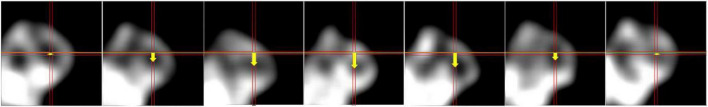
Coronal views of heart in an exhalation-inhalation sequence during one respiratory cycle. The respiratory period was 5 s while the heart beating period was 1 s. The cross hair marks the center of the left ventricle and the first frame represents the end-exhalation. The vertical shift of the myocardium is represented by an arrow.

Like in the case of 6D image reconstruction of the tensor product of basis functions (28) the gated cardiac reconstruction was able to delineate the deformation as well as the upward displacement of the heart due to respiration. In Figure 10 we show the cardiac displacement in an exhalation-inhalation sequence during one respiratory cycle. The crosshair marks indicate the center of the left ventricle while the fiduciary horizontal line is the reference drawn at the end-exhalation phase. The vertical arrow is the cardiac displacement from the reference line. Only 7 respiratory phases at time points 300.0, 301.0, 302.0, 302.5, 303.0, 304.0, and 305.0 s are shown. The first frame represents the end-exhalation.

**FIGURE 11:**
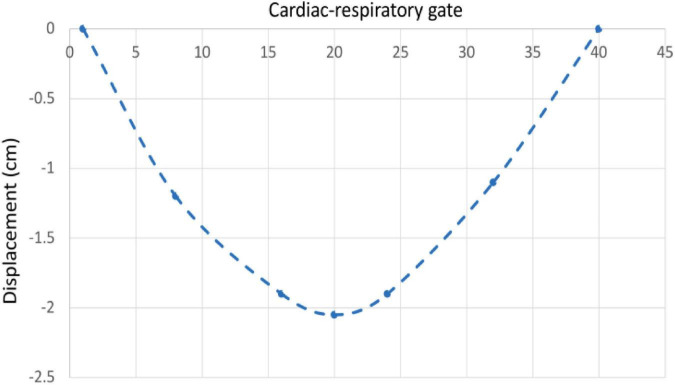
Liver displacement over a respiratory cycle.

In order to compare the magnitude of the displacement of the heart due to respiratory motion we plotted the center of the left ventricle as a function of the respiratory gates (the length of the vertical arrows in Figure 10) and is shown in Figure 11. The displacement is consistent with the displacement of the original phantom as shown in Figure 2.

**FIGURE 12:**
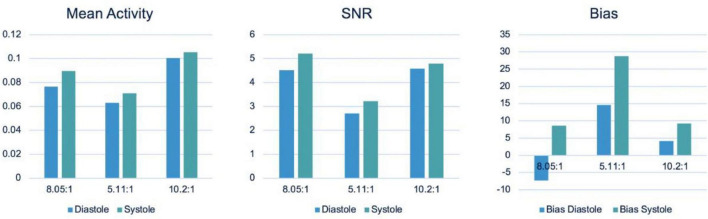
Performance evaluation: Mean Stabilized Activity (MSA), SNR and Bias for three different TBRs for myocardium and systole and diastole phases.

Figure 12 shows the detailed results of the statistical analysis of the reconstructed image for 3 different TBRs, namely Mean Stabilized Activity (MSA), SNR and Bias (%) at systolic and diastolic phases of the heart. The MSA and SNR were higher for a higher TBR while biases were improved for a higher TBR. There is no statistical difference between SNR at the TBRs = 8 and 10 (P = NS) while the SNR is decreased significantly for TBR = 5 compared to TBR = 8 (*P* < 0.001) with better bias-noise characteristics. This indicates that the TBR = 8 is sufficient to test the algorithm. Also, the variances were higher at TBR = 5 compared to 8 and 10.

### Comparison of 4D Reconstruction With Conventional Spatiotemporal Reconstruction

**FIGURE 13:**
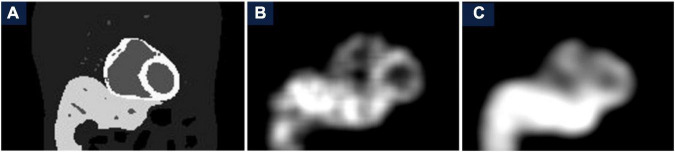
Comparison of gated reconstruction proposed in this work with conventional 4D spatiotemporal reconstruction without gating. Ground truth image **(A)** and the corresponding reconstructed images with the method proposed in this work with only 18 views per rotation **(B)** and with the conventional method with 120 views per rotation for the same data set **(C)** without gating.

The spatiotemporal 4D reconstruction provides information of the temporal changes of the radiotracer at each spatial point without specifics of the cardiac or respiratory motion. The reconstructed images with MC simulation were compared with the data obtained from the conventional spatiotemporal 4D reconstruction that has been implemented in our earlier publications in patient studies (12, 42). In these studies, for a TBR = 8 we binned data over each 3-degree view for a total of 120 views per rotation per detector head. Despite the under sampled data set with only 18 views per rotation, the image quality has significantly improved with the proposed method. In Figure 13 we show the comparison of the gated reconstruction (B) proposed in this work with conventional 4D spatiotemporal reconstruction without gating (C). The 4D conventional reconstruction showed image blurs due to respiratory motion while the gated reconstruction provided the information of cardiac and respiratory motion. Our reconstruction method also showed better resolved myocardial walls during diastole to systole as compared to the conventional ungated 4D spatiotemporal reconstructed image.

### LV Wall Non-uniformity and Diaphragm Displacement

**FIGURE 14:**
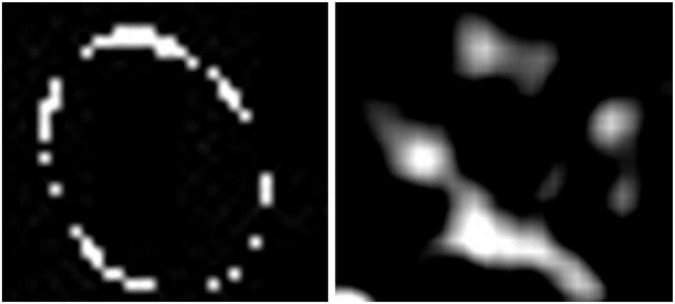
Effect of LV wall non-uniformity (Case 4): Comparison of the difference in LV wall thickness between the ground truth (left) and the reconstructed image.

Our MC simulation with 4D XCAT phantom is capable of simulating the heterogeneous myocardial wall thickness (Case 4) as well as variable diaphragm displacement (Case 5). We also simulated variable myocardial wall thickness to test our reconstruction method. In Figure 14 we show the difference between the reconstructed images with and without LV wall non-uniformity and compared with the ground truth derived from the original phantom (Figure 6). Despite of highly under-sampled data, the thinner myocardial wall regions are clearly delineated with higher intensity.

**FIGURE 15:**
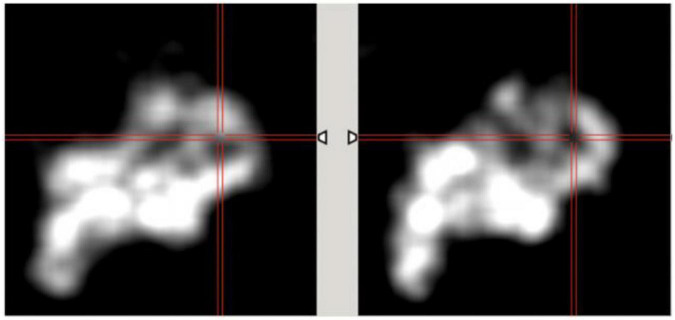
Effect of diaphragm displacement (Case 5). The images are shown at the interval of 0.5 s after full inhalation to show the myocardial displacement due to respiration for diaphragm displacement *D* = 1.5.

Figure 15 shows an example of diaphragm displacement with *D* = 1.5 cm (Case 5). As in Figure 10, the amplitude of the displacement approximately matched with the true displacement derived from phantom data. The images are shown after the end-inhalation to full exhalation of the respiratory phase.

### Estimation of Dynamical Quantitative Indices

**FIGURE 16:**
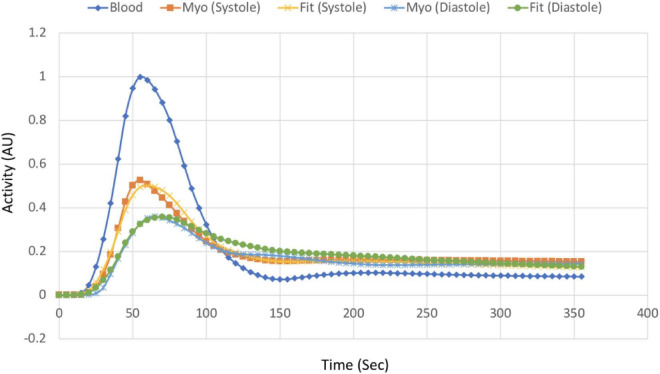
TACs of the myocardium at systole and diastole phase and corresponding fits along with the input function (LV blood pool) for Case 1 study. The data was fitted with the 1TCM for the simulation using LV blood pool as an input function.

In Figure 16 we show an example of TACs of the LV blood pool and total myocardium at systole and diastole for the Case 1 study (TBR = 8) and the corresponding fits using the 1TCM. Table 3 provides the results of the quantitative dynamic rate constants (K_1_ and k_2_) and V_L_ for Tc-99m tetrofosmin derived from the fits for different TBRs along with the corresponding ground truth values.

**TABLE 3:** Quantitative indices estimated using 1TCM at systole and diastole.

		K_1_ (ml/g/min)	k_2_ (1/min)	V_L_ (%)
Case 1	Systole	0.32	0.25	42
	Diastole	0.39	0.27	33
Case 2	Systole	0.28	0.23	45
	Diastole	0.31	0.26	34
Case 3	Systole	0.38	0.3	40
	Diastole	0.42	0.35	33
Ground truth		0.33	0.21	40

## Discussion

This study was designed to simultaneously model the changes in activity in the myocardium by incorporating cardiac and respiratory motion using a commonly available two-head SPECT system. This was accomplished by a continuous acquisition of the data during wash-in and wash-out of the radiotracer. Although the rotation was relatively slow (90 s per rotation per head), the projection views were tuned to capture the respiratory and cardiac gate along with the changes in radiotracer activity. By acquiring the data in a LM format that has the information of cardiac and respiratory phases while camera rotates continuously, the emitted photons were binned for a very short time duration (0.125 s) in each detector pixel. With no photon loss between detector consecutive positions, we were able to capture essentials of regional myocardial perfusion dynamics in a deformed tissue due to cardiac-respiratory motion. The method described in this work, though technically feasible, requires a very large number of views per rotation and thus a very small number of photons per pixel per view with significantly higher noise in the system and requires a more accurate reconstruction algorithm.

A general approach for cardiac motion compensation assumes the 3D configuration and relies on the fact that the activity in a given voxel is fixed. This is true in conventional (static SPECT) clinical acquisition where the data is acquired only after the tracer is fully stabilized in the myocardium. However, for dynamic acquisition the tracer activity changes significantly during wash-in and wash-out phase. Therefore, to calculate more accurate kinetic modeling parameters the temporal changes of the activity along with the cardiac-respiratory motion must be incorporated.

In this work, we extended our previous work and applied our 6D reconstruction algorithm to reconstruct the dynamic cardiac SPECT data with realistic Monte Carlo simulation acquired by a slowly rotating gamma camera. The term “slowly rotating camera” here refers to the rate of rotation that is comparable or smaller than the tracer kinetics i.e., the wash-in and wash-out rates of the given radiotracer.

A major issue of the dynamic acquisition with rotating SPECT system is the inconsistency of the data, as there are only two views at a given time. Inconsistency would not be a problem during the late phase of the tracer dynamics once the activity distribution stabilizes in the myocardium. Myocardial radiotracers such as Tc-99m sestamibi or Tc-99m tetrofosmin, which are usually utilized in clinical SPECT, generally stabilizes 20 min after the injection. But for the dynamic acquisition, this is particularly challenging during the early phase of the blood-myocardial wash-in and wash-out exchanges. This problem is much more difficult in SPECT than for ring detector systems such as dynamic cardiac PET (43). To address this issue, we have developed a 4D reconstruction algorithm (7, 39) to simultaneously solve a smooth time varying distribution of the tracer activity concentration in the myocardium using B-spline basis functions in both phantom and clinical patient studies (12), and extended the method to 6D by incorporating cardiac and respiratory motions (28).

In our previous algorithm (28), we used the tensor product of spatiotemporal basis functions (B-splines) and basis functions of the subspace of cardiac and respiratory phases (Gaussians) with synthetic data from the mathematical cardiac torso (MCAT) phantom generated by the forward projection of the cardiac torso using the same system matrix for a given detector configuration. This method had two major problems, mainly the computational complexity, as it requires the tensor products of 6 basis functions and the size of the system matrix which was assumed to be time independent. It should be noted that the system matrix for a cardiac-respiratory system is inherently time-dependent due to continuous motion of the heart and lungs. In order to simplify the problem, we decouple the cardiac and respiratory gates by translating the 6D problem into 4D while still capturing the cardiac and respiratory motions as well as tracer dynamics by acquiring the data in a continuous fashion. We used the more advanced 4D XCAT phantom (29) and Monte Carlo simulation to delineate a realistic clinical scenario of the myocardial perfusion imaging dynamics by incorporating cardiac and respiratory motion information.

We used the standard maximum likelihood expectation maximization (ML-EM) algorithm (44) for estimating the activity distribution. The ML-EM algorithm was successfully implemented in the past for modeling four-dimensional (4D) SPECT acquisition as well as for five-dimensional (5D) PET and SPECT acquisitions (45). The change of the radiotracer concentration in the myocardium is modeled using spatiotemporal cubic B-spline basis functions corresponding to each cardiac and respiratory gate. Spatiotemporal basis functions have been the basic building blocks to model the smooth variation of radiotracer distribution in space and time (39). Generally, the number of basis functions and their temporal extents (knots) can be optimized for an arterial input function. In all simulations we have used 9 basis functions optimized with the blood pool peak and wash-out rate in a patient’s TAC.

Our group has also investigated in some detail the L-mode acquisition without cardiac respiratory gating for dynamic cardiac SPECT (46). In our previous work with the Philips Precedence SPECT/CT dual-headed scanner we demonstrated that a 1 min infusion with a two-headed SPECT system with L-mode acquisition rotating 180° every 54 s can produce reliable measurements of blood pool and myocardial TACs. The present work was to investigate a dynamic cardiac acquisition with our current GE Infinia Hawkeye clinical scanner with H-mode acquisition. Both H-mode and L-mode acquisitions present challenges in acquiring dynamic data. The L-mode acquisition provides more consistent views in a shorter time interval, whereas H-mode provides less consistent views in a time interval but more opposing views in a shorter time interval with additional attenuation information. In both cases it is necessary to correct for the attenuation in the projections. The purpose of the dynamic acquisition with continuous camera rotation is to develop a model for tracer kinetics that can capture as many views as possible for a given tracer concentration to avoid tomographic inconsistencies. We utilize a continuous camera rotation scheme with B-splines that smooth out the irregularities coming from under-sampled data (inconsistency). We have not simulated L-mode acquisition in this work, but it is interesting to see how these two acquisition methods compare.

Counterintuitively, in Figure 12, the SNR for TBR = 8 in the systolic phase is higher than for TBR = 10 but they are not statistically significant. There are many confounding factors including selection of ROI that may have caused this discrepancy. For MC simulation, we already know the segmented organ information from the 4D XCAT phantom. We therefore did not need the additional segmentation information during simulation and reconstruction. However, for the reconstructed image analysis a better segmentation will delineate a clear myocardium boundary. In this work we used a manual segmentation that may have caused this error.

Despite the limited angle reconstruction and non-homogeneity in the myocardium due to inconsistency, our gated reconstruction method faithfully reproduces the K_1_ and k_2_ values as derived from the conventional method without gate and the results are very close to the ground truth. This method also enables us to differentiate these dynamic parameters during different phases of the heart, though we do not know the clinical significance of these differences at this time. The higher k_2_ value in the gated reconstruction compared to conventional ungated method seems to arise from the fact that we have simulated and fitted only up to 6 min of dynamic data out of a total of 20 min of acquisition in the patient study.

As described in our earlier publication (28), our method has some limitations on future clinical implementation. Our method requires a very large number of views and a sufficient number of cardiac-respiratory phases per rotation, with a small number of photons per pixel per view. This could significantly increase the noise in the system if the injected dose is small. For a sufficient number of photons per pixel, one has to increase the injection dose. Although this study was performed with realistic data generated by Monte Carlo simulation, it is still considered as a simulation. For example, even as we set different activities in different organs, only a few organs were considered in the simulations. There were significant liver scattering and background activities that were ignored in the simulation. Therefore, our results might be oversimplified. We did not test our method with real patient data because the current camera system does not provide gating information during a dynamic acquisition. A modification in clinical SPECT systems should be able to acquire dynamic cardiac and respiratory-gated data and this will be a future plan. We therefore believe that this method can be within the reach of clinical testing with real patient data.

## Conclusion

Our study demonstrated the viability of using continuous image acquisition method on a widely used clinical two-head SPECT system by eliminating the dead time between the angles at consecutive detector positions. The continuous image acquisition for dynamic scan using conventional gamma cameras can provide valuable information to myocardial perfusion imaging. Precise implementation of reconstruction algorithms, better segmentation techniques by generating images of different tissue types and background activity would improve the feasibility of the method in real clinical environment.

## Data Availability Statement

The raw data supporting the conclusions of this article will be made available by the authors, without undue reservation.

## Author Contributions

YH: MC simulation and manuscript writing. US: reconstruction and manuscript writing. GG and YS: conceptualize the problem, design simulation, manuscript writing, and review. All authors contributed to the article and approved the submitted version.

## Conflict of Interest

The authors declare that the research was conducted in the absence of any commercial or financial relationships that could be construed as a potential conflict of interest.

## Publisher’s Note

All claims expressed in this article are solely those of the authors and do not necessarily represent those of their affiliated organizations, or those of the publisher, the editors and the reviewers. Any product that may be evaluated in this article, or claim that may be made by its manufacturer, is not guaranteed or endorsed by the publisher.
